# Genetic Variants Detection Based on Weighted Sparse Group Lasso

**DOI:** 10.3389/fgene.2020.00155

**Published:** 2020-03-03

**Authors:** Kai Che, Xi Chen, Maozu Guo, Chunyu Wang, Xiaoyan Liu

**Affiliations:** ^1^ School of Computer Science and Technology, Harbin Institute of Technology, Harbin, China; ^2^ School of Electrical and Information Engineering, Beijing University of Civil Engineering and Architecture, Beijing, China; ^3^ Beijing Key Laboratory of Intelligent Processing for Building Big Data, Beijing, China

**Keywords:** genome-wide association studies, genetic variants, single nucleotide polymorphisms, minimum allele frequency, sparse group lasso

## Abstract

Identification of genetic variants associated with complex traits is a critical step for improving plant resistance and breeding. Although the majority of existing methods for variants detection have good predictive performance in the average case, they can not precisely identify the variants present in a small number of target genes. In this paper, we propose a weighted sparse group lasso (WSGL) method to select both common and low-frequency variants in groups. Under the biologically realistic assumption that complex traits are influenced by a few single loci in a small number of genes, our method involves a sparse group lasso approach to simultaneously select associated groups along with the loci within each group. To increase the probability of selecting out low-frequency variants, biological prior information is introduced in the model by re-weighting lasso regularization based on weights calculated from input data. Experimental results from both simulation and real data of single nucleotide polymorphisms (SNPs) associated with *Arabidopsis* flowering traits demonstrate the superiority of WSGL over other competitive approaches for genetic variants detection.

## Introduction

Since completion of the sequencing-based structural genome project, the focus of life science research has gradually shifted from determining the composition of DNA sequences to elucidating the function of identified genes. However, the greatest challenge of functional genomics is to determine the risk genes associated with complex diseases or traits among the huge amount of DNA sequences. Approximately, 90% of all gene fragments in any two individuals of almost all organisms are identical; thus, the fragments affecting individual characteristics, diseases, or traits only appear in a small range of sequences (Tenaillon et al., 2001; Reich et al., 2002). Polygenic recombination or mutation can cause individual differences in genome sequences, resulting in genetic polymorphism. Single nucleotide polymorphisms (SNPs) are the most common form of such genetic variation. Therefore, identification and characterization of SNPs help to discover the underlying causes of various diseases or variable traits and to develop new therapeutic strategies and targets for drug development or crop improvement.

The goal of genome-wide association studies (GWAS) is to elucidate the relationship between millions of SNPs and complex traits (Klein et al., 2005). A single-locus association approach is typically used in GWAS; however, the “polygenic theory” proposes that complex traits are controlled by the action of multiple SNPs together rather than by individual genes or variants (Dudbridge, 2016). Since the number of SNPs far exceeds the number of samples in a multi-loci association study, the “curse of dimensionality” becomes the main challenge of this type of analysis (Waddell et al., 2005). Many machine-learning algorithms have been widely used to overcome this limitation and facilitate investigating the association between traits with SNPs. Based on current approaches, association studies can be divided into two main categories: one based on feature selection (FS) and the other based on statistical machine learning with regularizing penalty.

FS is the process of selecting the most effective features among a set of original features so as to reduce the dimensionality of the dataset. There are two types of FS methods: the wrapper method as a dependent classifier (Hall and Smith, 1999), and the filter method as an independent classifier (Liu and Setiono, 1996). Typically, the wrapper and filter approaches are combined as the final selected method. When applying FS methods to GWAS, the SNPs are treated as the features, phenotypes are the labels, and the candidate SNPs are then selected according to their associations with phenotypes. Numerous FS methods have been applied in genetic association studies (Evans, 2010; Batnyam et al., 2013; Anekboon et al., 2014; Alzubi et al., 2017; An et al., 2017; Setiawan et al., 2018; Tsamardinos et al., 2019). For example, Evans (2010) combined two filter FS methods with classification methods in a machine-learning approach, and obtained strong association results. To further improve the accuracy of the selected SNPs, Batnyam et al. (2013) applied four popular FS approaches (Robnik-Šikonja and Kononenko, 2003; Liang et al., 2008; Seo and Oh, 2012; Lee et al., 2013) to select novel SNPs, which were then used to generate artificial features by applying a feature fusion method. Finally, the artificial features were classified by traditional classifiers. As an alternative combinational algorithm, Anekboon et al. (2014) proposed a correlation-based FS method as a filter to first select a portion of the SNPs, followed by a wrapper phase to sequentially feed each of these SNPs into k-nearest neighbor, artificial neural network, and Ridge regression classifiers. Alzubi et al. (2017) developed a hybrid FS method by combining conditional mutual information maximization and support vector machine-recursive feature elimination (SVM-RFE). An et al. (2017) used a hierarchical feature and sample selection framework to gradually select informative features and discard ambiguous samples in multiple steps to improve the classifier learning. Setiawan et al. (2018) firstly employed random forest algorithm to reduce the search space, then selected associated SNPs by sequential forward floating selection. Tsamardinos et al. (2019) applied p-values of conditional independence tests and meta-analysis techniques to select features, and made use of parallel techonology to increase the computing speed. Current methods based on FS have sufficient ability for selecting a candidate feature set. Nevertheless, it is important to use available biological information as prior knowledge in biocomputing. Since FS methods can only reflect the dataset itself, they are not suitable to screen features based on prior biological knowledge.

Regression models with penalty can also be used for GWAS. With this approach, the SNPs correspond to the independent variables, and phenotypes are mapped to dependent variables in the regression model. Since the number of SNPs typically far exceeds the number of samples, it is necessary to regularize the sparsity of coefficients in the regression model. As a representative example, the well-established lasso method proposed by Tibshirani (1996) can learn a sparse weight vector by penalizing the weight vector with a 1-norm loss while shrinking less important coefficients to zeros. Owing to this property, lasso and its extensions have been widely applied in the detection of genetic variants (Cao et al., 2014; Arbet et al., 2017; Tamba et al., 2017; Cherlin et al., 2018; Wang et al., 2019). For example, Cao et al. (2014) incorporated prior information in lasso to further increase the selection accuracy. Arbet et al. (2017) imposed a permutation method on lasso to improve the performance of the algorithm. Tamba et al. (2017) first reduced the number of SNPs to a moderate size, then used expectation maximization Bayesian lasso to detect the quantitative trait nucleotide (QTN). Cherlin et al. (2018) used lasso to explore the association between phenotype and SNP data and achieved good prediction. Wang et al. (2019) promoted a precision lasso that utilized regularization governed by the covariance and inverse covariance matrices of explanatory variables to increase sparse variable selection. However, SNPs (features) are generally found in groups, whereas lasso does not encourage sparsity between groups. Yuan and Lin, (2006) proposed the group lasso (GL) method, which sets a regularization of the sum of the ℓ_2_ norm onto groups that encourages only a few groups to be selected. The GL approach has also been successfully applied in GWAS (Li et al., 2015; Lim and Hastie, 2015; Gossmann et al., 2017; Du et al., 2018). Gossmann et al. (2017) extended sorted L1 penalized estimation (SLOPE) in the spirit of Group LASSO to handle group structures between the predictor variables. Du et al. (2018) proposed the SCCA with truncated L1 penalized and GL to improve the performance and effectiveness of discovering SNPs or QTs in imaging genetics. However, once a group is chosen, all of its comprising features are also selected, which is not compliant with the actual biological situation in which SNPs are distributed sparsely across the genome in only a few groups. Simon et al. (2013) developed sparse GL (SGL) that uses the ℓ_2_ penalty to select only a subset of the groups and the ℓ_1_ penalty to select only a subset of the variables within the group. Indeed, SGL has been widely applied in detecting genetic variants (Rao et al., 2015; Li et al., 2017; Samal et al., 2017; Guo et al., 2019). Samal et al. (2017) proposed a method based on SGL to identify phenotype associated extreme currents decomposed from metabolic networks data. Combined SGL with group-level graph structure, which takes advantages of gene-level priors to penalty the nucleotide-level sparsity to identify the risk SNPs. Guo et al. (2019) proposed a method that combined SGL and linear mixed model (LMM) for multivariate associations of quantitative traits, and it obtained a good power. Despite this improvement, the limitation of this method is that SGL selects sparse features within a group, but gives the same penalty for all features within the group. Consequently, this approach can easily result in swiping out low-frequency features that may play an important role in influencing phenotypes. To overcome this obstacle, it is important to assign different penalties to different features. Ideally, candidate SNPs should have a smaller penalty weight while others would have a larger penalty weight. In this way, candidate SNPs will stand out among the data more readily. To achieve this goal, we here propose a novel approach termed weighted SGL (WSGL) by introducing biological prior information for more accurate genetic variants detection. Specifically, we compute the minimum allele frequency (MAF) among a dataset of SNPs and use those values to reweight as the ℓ_1_ penalty of each SNP site, which can increase the chance of retaining low-frequency variants without loss of information. To validate this approach, we compared the performance of our model with simulation and real data against the three mainstream models discussed above.

## Materials and Methods

### Materials

#### Simulation Data

We used *Arabidopsis thaliana* data from Atwell et al. (2010), downloaded from https://github.com/Gregor-Mendel-Institute/atpolydb for the simulation. We used a quality control protocol on the original data. The SNPs are eliminated by the standard that Minor Allele Frequency (MAF) is < 0.01, the missing rate is > 0.05, or the allele frequencies are not in Hardy-Weinberg (*P* < 0.0001). After data preprocessing, we chose 200 genes on chromosome 1 covering a total of 1,993 SNPs. Twenty of these SNPs were chosen as the associated variants.

#### Real Data

The genotype information was the same as that obtained from the simulation data. Ten phenotypes were selected among the 107 reported. First, from chromosome 1 to 5, we chose the first 1,000 genes, which were sorted according to sequence length, including 49,962 SNPs. Second, we selected 19 genes containing 367 SNPs, which have been verified to be associated with flowering time in *Arabidopsis*. Thus, a total of 50,329 SNPs were analyzed in our experiments.

### Statistical Model and Methods

We first give a problem statement, followed by a brief overview of lasso and its extension for application in a genetic association study. Finally, we describe our new WSGL method.

Let *X* = (*x*_1_, *x*_2_,…, *x_n_*)*^T^* denote the *n* × *p* genotype matrix, where *n* is the number of samples and *p* is the number of genotypes. Let *Y* = (*y*_1_, *y*_2_,…, *y_n_*)*^T^* represent the *n* × 1 phenotype vector, containing the phenotype values of the *n* samples. We then establish a linear model between *X* and *y*:

(1) Y=Xβ+ϵ

where *β* = (*β*_1_, *β*_2_,…, *β _n_*)*^T^* is a *p* × 1 regression coefficients vector, and ϵ ∼*N*(0, 1).

#### Lasso and Its Extension for Association Mapping

Tibshirani (1996) proposed the popular lasso estimator,

(2) $$\underset{\beta }{\min}\frac{1}{2}\|y-X\beta {\|}_{2}^{2}+\lambda \|\beta {\|}_{1}$$

where *β* is the regression coefficients vector, and *x*, corresponding to the nonzero estimated coefficients in *β*, represents the candidate SNPs. ||*β|*|_1_ is the ℓ_1_ penalty item. λ is a regularization parameter, and its size determines the sparsity. When λ = 0, the lasso estimator is equivalent to ordinary least-squares regression.

However, the lasso applies to the situation in which the variables are independent of each other. For the situation in which the variables can be divided into *m* groups, Yuan and Lin (2006) proposed the GL estimator,

(3) $$\underset{\beta }{\min}\frac{1}{2}\|y-\overset{m}{\underset{l=1}{\sum }}X^{\left(l\right)}\beta^{\left(l\right)}{\|}_{2}^{2}+\lambda \overset{m}{\underset{l=1}{\sum }}\sqrt{p_{l}}\|\beta^{\left(l\right)}{\|}_{2}$$

where *m* is the group of variables, the first part is OLS, the second part is the sum of the ℓ_2_ penalty of the coefficients of each group, and λ is the regularization parameter. If the size of the group is 1, it will degenerate to the standard lasso.

The GL can generate a sparse in groups; however, the variables in a group are not sparse. To solve this problem, Simon et al. (2013) proposed the SGL,

(4) $$\underset{\beta }{\min}\frac{1}{2n}\|y-\overset{m}{\underset{l=1}{\sum }}X^{\left(l\right)}\beta^{\left(l\right)}{\|}_{2}^{2}+\left(1-\alpha \right)\lambda \overset{m}{\underset{l=1}{\sum }}\sqrt{p_{l}}\|\beta^{\left(l\right)}{\|}_{2}+\alpha \lambda \|\beta {\|}_{1}$$

where λ still controls the overall penalty and α determines the ratio between ℓ_1_ and ℓ_2_. When α = 1, it will be transformed into lasso, whereas when α = 0, it will be GL. SGL can either select the variables in a group-by-group manner, or screen the individual variables in the remaining groups.

### Our Method

With respect to the genetic association problem, the variables in a group have different effects on the independent variable. However, the SGL uses the same penalty coefficients for all variables, regardless of the relative importance among SNPs in the screened groups.

To tackle this problem, we introduce the prior information ω in the model to improve the statistical power, and propose the WSGL,

(5) $$\underset{\beta }{\min}\frac{1}{2n}\|y-\overset{m}{\underset{l=1}{\sum }}X^{\left(l\right)}\beta^{\left(l\right)}{\|}_{2}^{2}+\left(1-\alpha \right)\lambda \overset{m}{\underset{l=1}{\sum }}\sqrt{p_{l}}\|\beta^{\left(l\right)}{\|}_{2}+\alpha \lambda \|\omega \beta {\|}_{1}$$

The objective function in (5) is clearly convex; therefore, the optimal solution can be achieved by subgradient equations. Let$\hat{\beta }$be the optimal solution of WSGL. For group *k* = (1, 2,…, *m*), the solution ${\hat{\beta }}^{\left(k\right)}$ satisfies

(6) $$\frac{1}{n}X^{\left(k\right)T}\left(y-\overset{m}{\underset{l=1}{\sum }})X^{\left(l\right)}{\hat{\beta }}^{\left(l\right)}\right)=\sqrt{p_{k}}\left(1-\alpha \right)\lambda \mu^{\left(k\right)}+\alpha \lambda \omega^{\left(k\right)}\nu^{\left(k\right)}$$

where *µ* ^(^ *^k^* ^)^ and *v* ^(^ *^k^* ^)^ are subgradients of $\|{\hat{\beta }}^{\left(k\right)}{\|}_{2}$ and $\|{\hat{\beta }}^{\left(k\right)}{\|}_{1}$, respectively. According to Simon et al. (2013), $\mu^{\left(k\right)}={\hat{\beta }}^{\left(k\right)}/\|{\hat{\beta }}^{\left(k\right)}{\|}_{2}$ if *β* ^(^ *^k^* ^)^ ≠ 0; otherwise, || *µ* ^(^ *^k^* ^)^ ||_2_ ≤ 1. $\nu_{j}^{\left(k\right)}=sign\left({\hat{\beta_{j}}}^{\left(k\right)}\right)$ when ${\hat{\beta_{j}}}^{\left(k\right)}\neq 0$; otherwise, $\|\nu_{j}^{\left(k\right)}{\|}_{2}\leq 0$.

Following the analysis in Simon et al. (2013), the condition for ${\hat{\beta }}^{\left(k\right)}=0$ is

(7) $$\|S\left(X^{\left(k\right)T}\gamma_{\left(-k\right)}/n,\alpha \lambda \omega^{\left(k\right)}\right){\|}_{2}\leq \sqrt{p_{k}}\left(1-\alpha \right)\lambda$$

where$\gamma_{\left(-k\right)}=y-\sum_{l\neq k}X^{l}{\hat{\beta }}^{\left(l\right)}$is the partial residual of *y*, and *S* is defined as ${\left(S\left(a,b\right)\right)}_{j}=sign\left(a_{j}\right){\left(\left|a_{j}\right|-b_{j}\right)}_{+}$.

If ${\hat{\beta }}^{\left(k\right)}\neq 0$, the subgradient condition for $\beta_{i}^{\left(k\right)}$ becomes

(8) $$\frac{1}{n}X_{i}^{\left(k\right)T}\left(y-\overset{m}{\underset{l=1}{\sum }}X^{\left(l\right)}{\hat{\beta }}^{\left(l\right)}\right)=\sqrt{p_{k}}\left(1-\alpha \right)\lambda \frac{{\hat{\beta_{i}}}^{\left(k\right)}}{\|{\hat{\beta }}^{\left(k\right)}{\|}_{2}}+\alpha \lambda \omega_{i}^{\left(k\right)}\nu_{i}^{\left(k\right)}$$

This is satisfied for ${\hat{\beta }}^{\left(k\right)}=0$, if $\left|X_{i}^{\left(k\right)T}\gamma_{\left(-k,i\right)}\right|\leq n\alpha \lambda \omega$, where $\gamma_{\left(-k,i\right)}=\gamma_{\left(-k\right)}-\sum_{j\neq i}X_{j}^{\left(k\right)}{\hat{\beta }}^{\left(k\right)}$ is the partial residual of *y*.

When $\beta_{i}^{\left(k\right)}\neq 0$, we can get

(9) $${\hat{\beta }}_{i}^{\left(k\right)}=\frac{S\left(X_{i}^{\left(k\right)T}\gamma_{\left(-k,i\right)}/n,\alpha \lambda \omega \right)}{X_{i}^{\left(k\right)T}X_{i}^{\left(k\right)}/n+\left(1-\alpha \right)\lambda /\|{\hat{\beta }}^{\left(k\right)}{\|}_{2}}$$

For each locus, MAF indicates, to some degree, its rareness. The MAF of low-frequency variants is usually small, so the associated low-frequency variants are more susceptible to sparsity regularization than other common variants. With normal sparse group lasso, the pressure of being zeroed out on each locus within the same group is equally high. In this case, those low-frequency variants are more likely to be excluded during the process. So selection of an appropriate weight can help to filter out more accurate candidate low-frequency variants.

There are several approaches for deciding the weights. For example, a small penalty can be assigned to the loci in known susceptibility genes to ensure including them into the model. Alternatively, the weights can be dependent on the MAF. For a dataset including both low-frequency and common variants, low-frequency markers are assigned smaller weights to compensate for their low frequencies. Here, we assign each locus a weight as follows: $weight=2\sqrt{MAF\left(1-MAF\right)}$. Each weight ω*_i_* is calculated in advance, which contains genotypes and biological explanations. The importance of the *i*th variable can be adjusted by the weight ω*_i_*. Thus, to choose a locus, we can give it a relatively small penalty weight. Conversely, a larger weight can be assigned to exclude a locus. If ω*_i_* = 1, our model will be transformed to the SGL. Moreover, it is important to select an optimal regularization parameter λ, as a larger λ will generate a sparser result. For the present model, we chose cross-validation to select the optimal λ.

A brief algorithmic description of our method is shown in **Algorithm 1**. Let *n* represent the number of samples and *p* be the number of genotypes. The time complexity of subgradient step in each iteration is *O*(*np*). In real data, *p* is usually supposed to be large, resulting in comparatively high time complexity. Therefore, in genome-wide association analysis, we suggest to analyze chromosomes individually for huge genome.

**Algorithm 1:** Parameter estimation for weighted sparse group lasso.

Input: Genotype *X*, phenotype *y* ratio α, regularization hyperparameter λ
**Output:** Estimated $\hat{\beta }$
1: calculate $\omega =2\sqrt{MAF\left(1-MAF\right)}$;
2: **while** not converge **do**
3: **for** *k* from 1 to *number_of_groups* **do**
4: **for** *i* from 1 to *length_of_groups* (*k*) **do**
5: update ${\hat{\beta }}_{i}^{\left(k\right)}$using equation (9);
6: return $\hat{\beta }$;

### Performance Measurements

For performance evaluation of the new model, we treat the loci detection as a binary classification under class imbalance, in which associated loci are assigned the label 1, and all others are assigned the label 0. The testing frequency of each locus is then regarded as the predicted probability for label 1. The receiver operating characteristic (ROC) curve and the area under the precision-recall curve (AUPR) are typically used for performance assessments. The ROC curve is plotted based on the sensitivity and specificity, whereas AUPR is generated based on the precision and recall. In our problem, the number of variants is significantly lower than the number of all loci, resulting in an imbalanced dataset. In the ROC curve, the false positive rate cannot descend greatly when the true negative is huge. However, the AUPR is sensitive to false positive. Considering these factors, we chose the AUPR as the performance metric for this purpose.

## Results and Discussion

### Experiments on Simulation Data

For assessing the performance of WSGL in selecting candidate SNPs associated with a trait of interest, its performance was compared with lasso, GL, and SGL. Two parameters needed to be controlled in this experiment: α, which is the proportion of ℓ_1_ and ℓ_2_ loss in SGL, and λ, which is the coefficient of the entire regularization term and influences the sparsity. We set α to 0.95. Based on the results of cross-validation, λ was set to 0.09.

**Figure 1:**
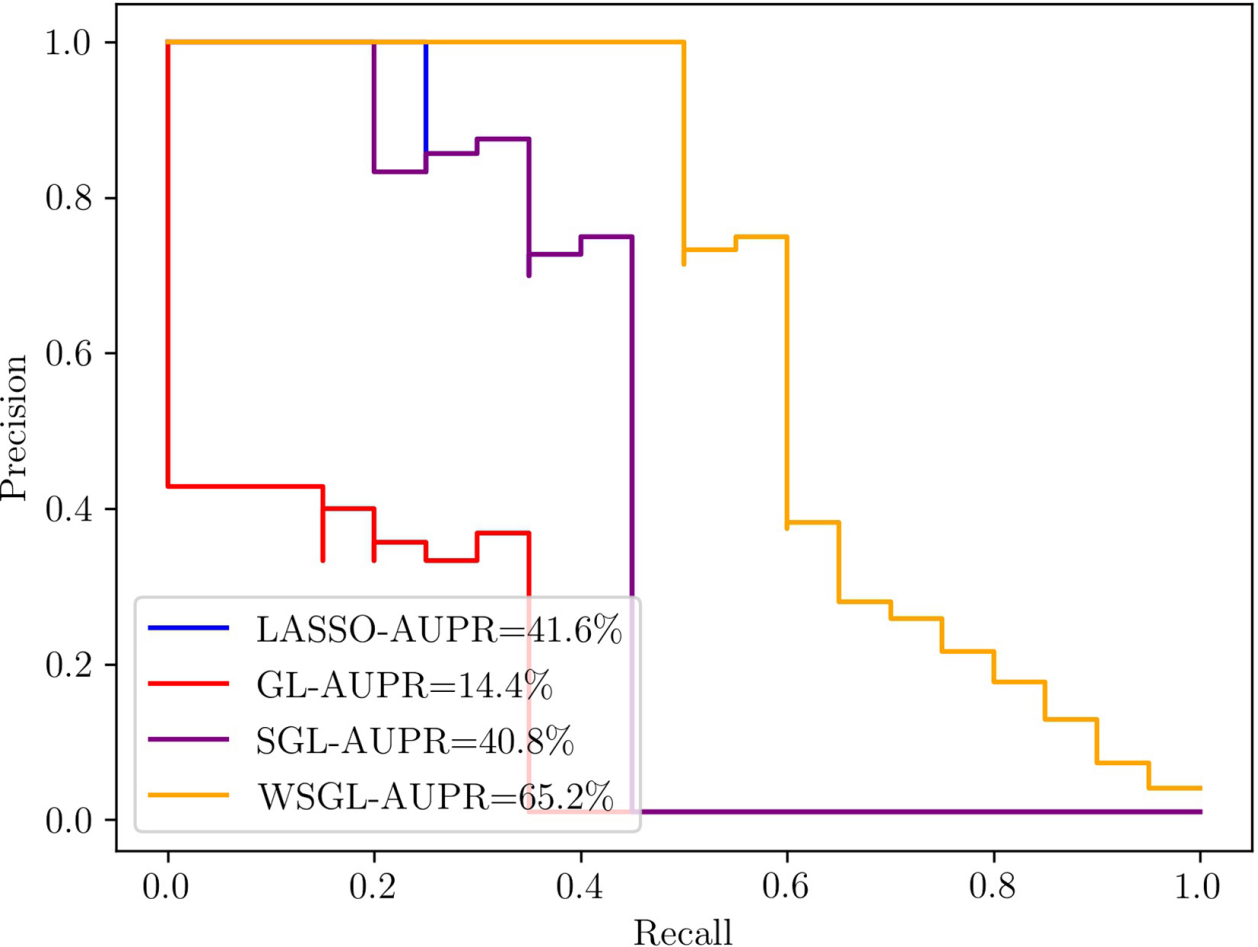
Precision-recall (PR) curves of WSGL and the other methods.

**Figure 1** shows the results of the four methods with the simulation data, which clearly exhibits the superior performance of WSGL. The AUPR of WSGL is 0.652, which outperformed lasso by 23.6%, GL by 50.8%, and SGL by 24.4%. Lasso uses ℓ_1_ to guarantee the sparsity of selected SNPs, but does not consider the group information; therefore, the candidate SNPs may be selected from all groups equally. Although GL imposes group information on the model, it still lacks sparsity constraint within the group, which does not correspond with the biological assumption that only a small number of candidate SNPs are contained in a small number of groups. SGL considers the sparsity between and within groups, but can still easily exclude important SNPs with a lower MAF. By introducing biological information to adjust the penalty of SNPs in the selected groups, WGSL places less weight on the low-frequency variants and thus increases their chance of being kept out. Despite its simplicity, the simulation results demonstrated the effectiveness of this approach for screening out important SNPs.

**Figure 2:**
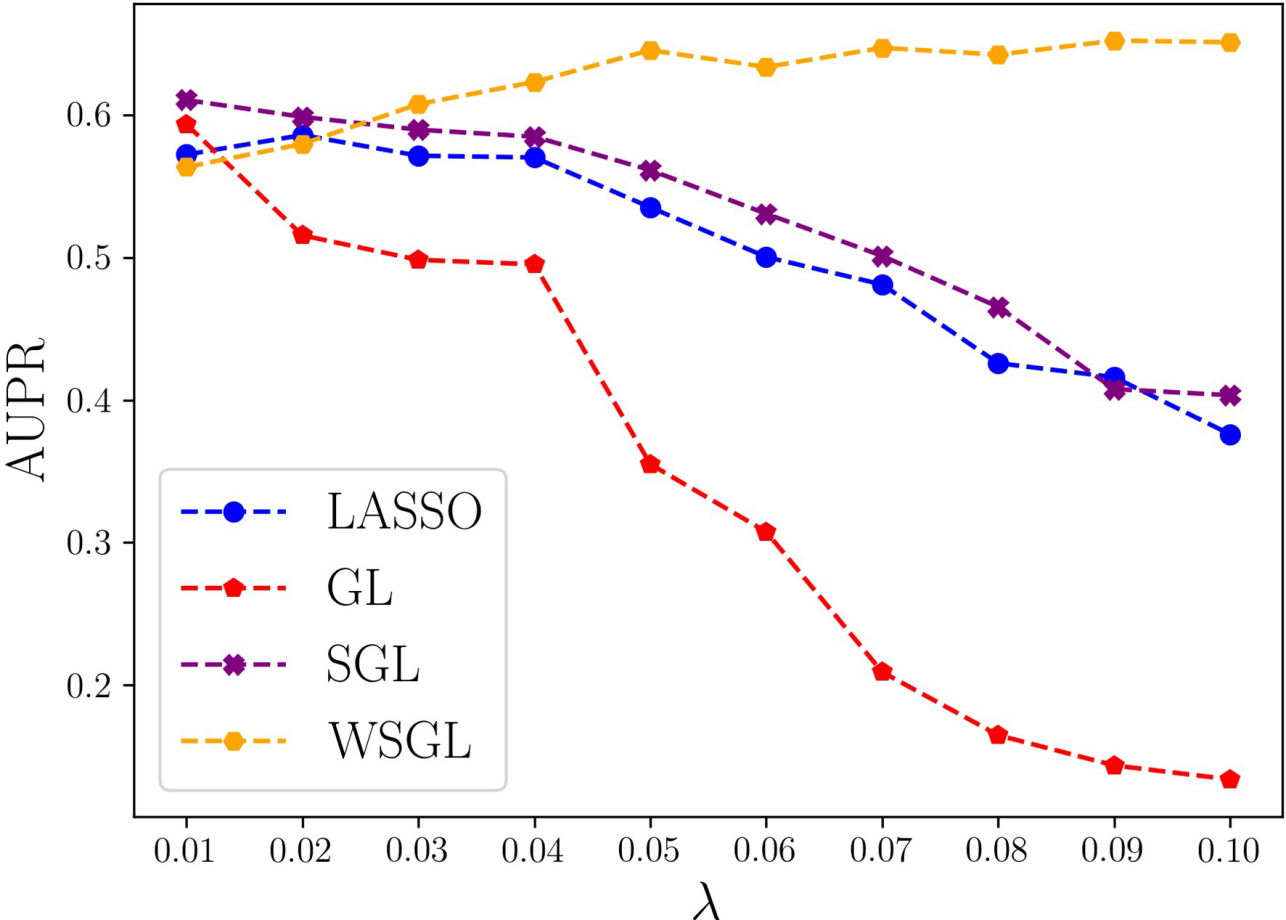
Precision-recall (PR) curves of WSGL and the other methods for varying λ.

To further compare the performance of the four algorithms, we computed their AUPR values by fixing α at 0.95 and varying λ from 0.01 to 0.1 by steps of 0.01. As shown in **Figure 2**, with smaller λ, the model shows lower sparsity. When λ is 0.01 or 0.02, the model will include more SNPs, which may include more non-candidate SNPs that would cause a high false positive rate. Conversely, as λ increases, the number of selected SNPs decreases, which might result in the loss of some candidate SNPs, leading to a low TP rate. However, WSGL will include more candidate low-frequency loci by introducing prior knowledge to adjust the weight. Accordingly, WSGL keeps the highest position starting from λ = 0.03. When λ increases from 0.02 to 0.05, the AUPR of WSGL increases significantly from 58% to 64.5%, whereas the AUPR of lasso decreases from 59.2% to 53.2%, and that of SGL decreases from 59.9% to 56.1%. Surprisingly, the AUPR of GL decreases even more sharply from 51.1% to 34.1%. When λ reaches 0.05, the AUPR of WSGL tends to be stable, and the peak of 65.2% occurs at λ = 0.09. The AUPR of both lasso and SGL gradually decreases, and finally drops to around 40%. When λ is 0.1, the AUPR of GL drops to 13.3%. These results were consistent with our expectation that the performance of WSGL would be the best, SGL would perform better than lasso, and GL would show the worst performance overall.

### Experiments on Real Data

To verify the ability of WSGL to detect candidate SNPs, we compared the performance of the four models using *Arabidopsis* flowering time data with known genetic associations. The dataset included 10 different phenotypes, FT10, FT16, FT22, LD, LDV, SD, SDV, LN10, LN16, and LN22, and the descriptions of the 10 phenotypes are shown in **Table 1**. We analyzed the associated number of genes covered by 100 SNPs with top probabilities of being target loci.

**Table 1:** Description of the 10 flowering related phenotypes in *A.thaliana* in real data application.

Phenotype	Accessions	Phenotype description	Growths conditions	Phenotype scoring
LD	167	Days to flowering time (FT) under Long Day (LD) and Short Days (SD) +/− vernalization	18°C 16-h daylight	Number of days following stratification to opening of the first flower. The experiment was stopped at 200d, and accessions that had not flowered at the point were assigned a value of 200.
LDV	168		18°C 16-h daylight, vernalized (5wks 4)	
SD	162		18°C 16-h daylight	
SDV	159		18°C 16-h daylight, vernalized (5wks 4)	
FT10	194		10°C 16-h daylight	
FT16	193		16°C 17-h daylight	Plants were checked bi-weekly for presence of first buds, and the average flowering time and average leaf number of four plants of the same accession at each temperature were collected.
FT22	193	Flowering time (FT) and leaf number at flowering time (LN)	22 °C 18-h daylight	
LN10	177		10 °C 19-h daylight	
LN16	176		16°C 20-h daylight	
LN22	176		22°C 21-h daylight	

As shown in **Table 2**, WSGL could link more candidate genes with phenotypes FT10, FT16, FT22, LD, SD, and SDV. In particular, WSGL demonstrated excellent performance for FT10, not only by selecting less groups but also by including less SNPs within each group, and the ratio of candidate genes was 23.08%. By contrast, the ratios of candidate genes were 4.65%, 9.09%, and 5.13% for lasso, GL, and SGL, respectively. For phenotypes FT16, FT22, LD, SD, and SDV, WSGL still achieved the best detection performance. However, unexpectedly, the GL model obtained better results for the first four phenotypes. We consider that this may be due to the specific distribution of loci in the dataset. In cases for which most or all of the candidate objects are located in only one group, GL will apparently show a good result. By contrast, all four methods could link all six genes with LDV, LN10, LN16, and LN22. This surprising result may reflect the strong association between the selected SNPs and these phenotypes, which is highly discriminable. Nevertheless, this assessment demonstrated that our new weighted method achieves the best performance overall, highlighting the importance of considering prior biological information for selection of candidate SNPs.

**Table 2:** Summary of four methods associations found in real data.

Phenotype	Method	Number of genes covered by top 100 SNPs	Number of genes in the 19 genes	Ratio of candidate genes
FT10	Lasso	86	4	4.65%
	GL	66	6	9.09%
	SGL	78	4	5.13%
	WSGL	26	6	23.08%
FT16	Lasso	76	8	10.53%
	GL	62	8	12.9%
	SGL	64	7	10.94%
	WSGL	67	10	14.93%
FT22	Lasso	78	7	8.79%
	GL	72	7	9.72%
	SGL	77	6	7.79%
	WSGL	71	9	12.68%
LD	Lasso	81	9	11.11%
	GL	67	9	13.43%
	SGL	73	11	15.07%
	WSGL	74	12	16.22%
LDV	Lasso	6	6	–
	GL	6	6	–
	SGL	6	6	–
	WSGL	6	6	–
SD	Lasso	78	5	6.41%
	GL	70	5	7.14%
	SGL	79	6	7.59%
	WSGL	77	6	7.79%
SDV	Lasso	84	1	1.19%
	GL	66	1	1.52%
	SGL	78	2	2.56%
	WSGL	72	2	2.78%
LN10	Lasso	6	6	–
	GL	6	6	–
	SGL	6	6	–
	WSGL	6	6	–
LN16	Lasso	6	6	–
	GL	6	6	–
	SGL	6	6	–
	WSGL	6	6	–
LN22	Lasso	6	6	–
	GL	6	6	–
	SGL	6	6	–
	WSGL	6	6	–

## Conclusion

We proposed a method named weighted sparse group lasso (WSGL) to improve the detection of genetic variants. WSGL incorporates the ℓ_1_ penalty, ℓ_2_ penalty, and prior biological knowledge into a single linear regression model, and then uses SGL to either select or clear out all SNPs in a group potentially associated with a phenotype of interest. To screen candidate low-frequency variants, we introduced the MAF as the weight to re-scale each element for calculating ℓ_1_ loss. In addition, WSGL can detect meaningful associations with more accuracy compared to available methods, which conforms with the general assumption that complex traits are affected by a few SNPs in a few genes. Experiments with both simulation and real data of SNPs related to the flowering time of *A. thaliana* demonstrated the effectiveness of our approach.

## Data Availability Statement

We used *Arabidopsis thaliana* data from Atwell et al. (2010), downloaded from https://github.com/Gregor-Mendel-Institute/atpolydb for the simulation.

## Author Contributions

Conceptualization: KC. Formal analysis: KC. Funding acquisition: MG, CW, and XL. Methodology: KC. Validation: KC and XC. Writing—original draft: KC and XC. Writing—review and editing, KC, MG, CW, and XL.

## Funding

This work was supported by the National Natural Science Foundation of China (Grant Nos. 61571163, 61532014, 61671189, 61872114, and 61871020) and the National Key Research and Development Plan of China (Grant No. 2016YFC0901902).

## Conflict of Interest

The authors declare that the research was conducted in the absence of any commercial or financial relationships that could be construed as a potential conflict of interest.
